# Effectiveness and safety of 3 and 5 day courses of artemether–lumefantrine for the treatment of uncomplicated falciparum malaria in an area of emerging artemisinin resistance in Myanmar

**DOI:** 10.1186/s12936-018-2404-4

**Published:** 2018-07-11

**Authors:** Kyaw Myo Tun, Atthanee Jeeyapant, Aung Hpone Myint, Zwe Thiha Kyaw, Mehul Dhorda, Mavuto Mukaka, Phaik Yeong Cheah, Mallika Imwong, Thaung Hlaing, Thar Htun Kyaw, Elizabeth A. Ashley, Arjen Dondorp, Nicholas J. White, Nicholas P. J. Day, Frank Smithuis

**Affiliations:** 1 Defence Services Medical Academy, Yangon, Myanmar; 2 Myanmar Oxford Clinical Research Unit, Yangon, Myanmar; 3 Centre for Tropical Medicine and Global Health, Nuffield Department of Clinical Medicine, University of Oxford, Oxford, UK; 4 Mahidol-Oxford Tropical Medicine Research Unit, Faculty of Tropical Medicine, Mahidol University, Bangkok, Thailand; 5 Medical Action Myanmar, Yangon, Myanmar; 6 Worldwide Antimalarial Resistance Network (WWARN), Asia Regional Centre, Bangkok, Thailand; 7 Department of Molecular Tropical Medicine and Genetics, Faculty of Tropical Medicine, Mahidol University, Bangkok, Thailand; 8 Department of Health, Ministry of Health and Sports, Naypyidaw, Myanmar

**Keywords:** *Plasmodium falciparum*, Artemether–lumefantrine, Artemisinin resistance, kelch13 mutation, Myanmar

## Abstract

**Background:**

Artemisinin resistance in *Plasmodium falciparum* has emerged and spread in Southeast Asia. In areas where resistance is established longer courses of artemisinin-based combination therapy have improved cure rates.

**Methods:**

The standard 3-day course of artemether–lumefantrine (AL) was compared with an extended 5-day regimen for the treatment of uncomplicated falciparum malaria in Kayin state in South-East Myanmar, an area of emerging artemisinin resistance. Late parasite clearance dynamics were described by microscopy and quantitative ultra-sensitive PCR. Patients were followed up for 42 days.

**Results:**

Of 154 patients recruited (105 adults and 49 children < 14 years) 78 were randomized to 3 days and 76 to 5 days AL. Mutations in the *P. falciparum* kelch13 propeller gene (*k13*) were found in 46% (70/152) of infections, with F446I the most prevalent propeller mutation (29%; 20/70). Both regimens were well-tolerated. Parasite clearance profiles were biphasic with a slower submicroscopic phase which was similar in *k13* wild-type and mutant infections. The cure rates were 100% (70/70) and 97% (68/70) in the 3- and 5-day arms respectively. Genotyping of the two recurrences was unsuccessful.

**Conclusion:**

Despite a high prevalence of *k13* mutations, the current first-line treatment, AL, was still highly effective in this area of South-East Myanmar. The extended 5 day regimen was very well tolerated, and would be an option to prolong the useful therapeutic life of AL.

*Trial registration* NCT02020330. Registered 24 December 2013, https://clinicaltrials.gov/NCT02020330

**Electronic supplementary material:**

The online version of this article (10.1186/s12936-018-2404-4) contains supplementary material, which is available to authorized users.

## Background

Artemisinin resistance in *Plasmodium falciparum* has emerged in South-East Asia and now extends over a large area of the Greater Mekong sub-region from the coast of Vietnam to the Eastern border of India [1–6]. Artemisinin resistance results in delayed parasite clearance leaving a larger residual number of parasites after a 3-day artemisinin-based combination therapy (ACT) course for the slowly eliminated partner drug to remove [7, 8]. This increases the probability that some parasites will survive to recrudesce. Artemisinin resistance has been followed by partner drug resistance in the eastern Greater Mekong sub-region, and this has resulted in a precipitous decline in the efficacy of dihydroartemisinin–piperaquine [4, 9]. Similar observations have been made along the Thai-Myanmar border where, after artemisinin resistance was established, mefloquine resistance reappeared quickly, and treatment failures to artesunate–mefloquine increased substantially [2].

New therapies are needed to treat artemisinin-resistant malaria, but there are currently very few options. Novel compounds, such as cipargamin [KAE609], artefenomel [OZ439] and KAF156 have shown promising results in phase 2 trials but are still under development [10–12]. In the absence of new drugs, strategies to counter the loss of ACT efficacy rely on adapted regimens of existing artemisinin-based combinations to optimize efficacy against resistant parasite strains, such as longer courses or triple drug combinations [1, 4, 13].

Artemether–lumefantrine (AL) is the first-line ACT in Myanmar. Artemether is mainly biotransformed to dihydroartemisinin [14, 15], the active metabolite, which is eliminated from the body very rapidly. Most of the infecting parasite biomass in acute malaria is cleared in the first two drug exposed cycles by the artemether and dihydroartemisinin components [7, 8]. The lumefantrine concentrations at the end of the treatment course are responsible for eliminating the residual parasites in the third and subsequent asexual cycles. Absorption of lumefantrine varies widely between individuals [14–19] and is dose limited [20]. Although AL is generally highly efficacious, children [17, 21] and pregnant women [19] are at higher risk of treatment failure. The lumefantrine plasma area under the concentration curve (AUC) *versus* time, or its surrogate the day 7 concentration, is the principal determinant of cure in acute falciparum malaria [15, 16]. When AL was evaluated first it was shown that splitting the six dose regimen over 5 days without increasing the total dose improved lumefantrine exposure and the efficacy of AL [15, 22]. Co-administration with a small amount of fat also improved bioavailability [23]. For patient groups with sub-optimal cure rates predictive modeling has suggested that both an increased dose and increased duration of treatment (a twice-daily regimen given for 5 days) are needed to increase cure rates [19]. This provides both an additional asexual cycle of artemether exposure and it increases the area under the plasma lumefantrine concentration–time curve (AUC) significantly.

Failure of AL in falciparum malaria has not been reported in Myanmar to date despite the emergence of artemisinin resistance and the decline in susceptibility to mefloquine, which shares resistance mechanisms with lumefantrine [2], but recent data are few and the experience from neighbouring countries indicates the need for frequent reevaluation. The hypothesis we intended to test was that prolonging the length of AL treatment could reduce the risk of treatment failure. This was done by conducting a two-way randomized trial to compare the tolerability, safety and effectiveness of a 5-day regimen of artemether–lumefantrine (AL5) with that of the standard 3-day regimen (AL3).

## Methods

### Study area and population

This study was conducted between November 2013 and February 2015 in two remote village tracts in Kyainseikgyi Township in Kayin State. Kayin state is situated in Southeast Myanmar, along the Myanmar-Thailand border. In 2012, Medical Action Myanmar, an international NGO, started malaria control activities in villages in this township through a network of 114 Village Health Volunteers (VHV), who tested all fever patients for malaria at community level with a rapid diagnostic test (RDT). Falciparum malaria was routinely treated with 3 days artemether–lumefantrine and a single gametocytocidal dose of primaquine according to national policy. In 2012–2013 these VHV tested 52,720 patients with a malaria RDT of whom 7541 (14.3%) tested positive; 4001 for *P. falciparum* (including mixed infections) and 3540 for *Plasmodium vivax*. Two VHV were trained to make blood-smears and to follow up patients with falciparum malaria for 3 days after treatment with AL. Of 78 patients followed up 11 (14%) were still positive by microscopy on day 3. Another study in this area has reported *k13* mutations in 48% of 42 patients studied [3].

### Study procedures

Patients with fever (tympanic temperature > 37.5 °C) or a history of fever, aged between 6 months and 65 years, with microscopy confirmed uncomplicated falciparum or mixed malaria, were invited to take part in the study if they had a parasite count between 80 (≥ 5/500 WBC on a thick film) and 175,000 asexual parasites per mm^3^. The main exclusion criteria were signs of severe or complicated malaria [18], haemoglobin less than 5 g/dL at enrolment, artemether–lumefantrine treatment in the preceding 28 days, known hypersensitivity to artemisinins, previous splenectomy and girls aged between 12 and 18 years (according to national ethics committee guidelines). Written informed consent was obtained from participants or parents/guardians in the case of children. The Oxford Tropical Research Ethics Committee (UK) and the Department of Medical Research Ethics Committee (Myanmar) approved the study protocol.

### Anti-malarial treatment

Patients were randomized in blocks of 12 to one of four treatment arms as follows:

1. 3 days AL (Coartem^®^) at standard doses twice daily
2. 3 days AL plus a one gram capsule of fish oil (Blackmores^®^)
3. 5 days AL in standard doses twice daily
4. 5 days AL plus a one gram capsule of fish oil (See Additional file 1).

A single dose of primaquine (0.25 mg/kg) was given to all patients on the 1st day of treatment. The initial AL treatment dose was given under supervision. All subsequent doses were given to the patient to be taken at home. The importance of taking these drugs, even when the symptoms had subsided, was explained clearly. At initial treatment, if the patient vomited within 30 min, the full dose was repeated. If vomiting occurred after 30 min but within an hour, half the dose was repeated.

### Monitoring

On enrolment, blood was taken for DNA extraction and full length sequencing of *Pfkelch13*. Patients were then seen on days 3, 5, 6 and 7, when 3 mL (1 mL from children ≥ 6 months < 5 years old) of venous blood was taken for parasite DNA quantitation with uPCR [24] (see Additional file 1).

Patients were then followed up weekly on days 14, 21, 28, 35 and 42 for temperature measurement, physical examination, blood smear and haemoglobin measurement (day 28 only). A symptom questionnaire including a list of specific questions related to adverse events was completed at each visit. Patients presenting again to the clinic with a microscopy confirmed *P. falciparum* or mixed infection within 7–42 days of follow up were treated with dihydroartemisinin–piperaquine and had a capillary blood sample collected onto filter paper for PCR genotyping to distinguish recrudescent from new infections [25].

After the first 39 patients had been enrolled, the uPCR collection time points were changed based on new information from another trial, which showed that 75% of patients with falciparum malaria still tested positive for parasite DNA with uPCR, on day 9 after ACT [26]. To characterize the late phase of parasite clearance and to increase the chances of detecting any differences between the two treatment arms, it was decided to drop the day 5 and day 6 samples and to add samples on day 14 and day 21. These changes were approved by the relevant ethics committees. Paired samples collected from patients with recurrent parasitaemia during follow up were genotyped to determine whether it was a new or recrudescent infection using three polymorphic markers: *msp1*, *msp2* and *glurp* [25].

### Statistical analysis

In order to detect a 20% difference in *P. falciparum* positivity in the 1st week detected by uPCR method or a difference in cure rates from 10 to 30% with 95% confidence and 80% power, 75 patients were required in each treatment arm. Data were entered into a web-based database Macro, (InferMed). Data cleaning and analysis were done using Stata14 (StataCorp) and GraphPad Prism 6 (GraphPad Software Inc.). Data were analysed by Student’s t-test, Wilcoxon-rank sum test and chi-squared test as appropriate. Survival data were analysed using the Kaplan–Meier method and Cox regression. Logistic regression was used to examine the relationship between treatment outcomes and mutation genotypes. A 5% significance level was used for all statistical tests.

## Results

A total of 1311 patients were screened for eligibility criteria and 154 (105 adults and 49 children < 14 years) patients were enrolled (see Additional file 1). There were three protocol violations which were inadvertent enrollment of three patients with parasite density in excess of the upper threshold stated in the inclusion criteria (two in the AL3 and one in the AL5 arm). All the treatments were well tolerated and all patients responded satisfactorily. Early discontinuation occurred in 13 patients; six patients withdrew consent, and seven patients were lost to follow up. Baseline characteristics are shown in Table 1. There were no significant differences between the two treatment arms.

**Table 1:** Baseline characteristics of the patients by treatment arm

Variables	Unit	Treatment arm		Total
		AL3	AL5	
Gender (male)	n/N (%)	48/78 (61.5)	48/76 (63.1)	96/154 (62.3)
Age	Year	20 (11–31)	18 (10–29.5)	19 (11–30)
BMI	Kg/m^2^	19.1 (16.8–21.1)	18.4 (15.8–20.2)	18.6 (16.2–20.8)
Parasite count (geometric mean, 95% CI)	/µL	3084 (1752–5430)	2140 (1269–3610)	2575 (1757–3775)
Gametocytaemia on admission	n/N (%)	17/78 (21.8)	16/76 (21.1)	33/154 (21.4)
Mixed infection	n/N (%)	1/78 (1.3)	2/76 (2.6)	3/154 (1.9)
Haemoglobin	g/dL	12.2 (11.1–13.7)	12.2 (10.8–13.4)	12.2 (10.9–13.4)

Data are presented as median (IQR) unless otherwise indicated

### Therapeutic responses-

Overall 20% (31/154) of all patients were still parasitaemic by microscopy on day 3 after starting treatment: 23% (18/78) of the AL3 treatment arm and 17% (13/76) in the AL5 treatment arm. On day 5, two of 78 patients were still positive after AL3 and 0 (0/76) after AL5. By day 7 all patients were microscopy negative.

Only two patients had a recurrence of *P. falciparum* parasitaemia detected by microscopy during follow up, both after AL5. A 6 years old child who was symptomatic on day 28, and a 24 year old male who was asymptomatic on day 42. Neither patient received fish oil. DNA extraction from blood samples taken from both patients was unsuccessful so genotyping to confirm recrudescence or reinfection could not be carried out. Therefore, the recurrence rate was 2.9% (2/70, 95% CI 0.35–10) in the AL5 arm and 0% (0/70, 95% CI 0–5.1) in the AL3 arm).

Three patients had mixed infections with *P. vivax* at baseline. These *P. vivax* infections were all cleared by day 3. Four patients, one of whom had a mixed infection detected at presentation, had a *P. vivax* recurrence (presumed relapse) during follow up; 2 at day 35 and 2 at day 42 (1 after AL3 and 3 after AL5).

#### Late phase parasite clearance

**Fig. 1:**
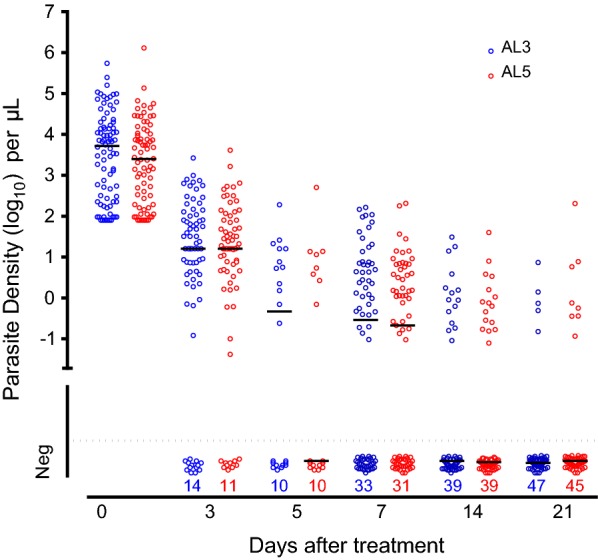
Clearance of *P. falciparum* parasitaemia assessed by quantitative uPCR. One circle represents one patient, red circles are treated by AL5, blue circles are treated by AL3

The profile of submicroscopic late phase parasite clearance estimated by uPCR showed a biphasic pattern in many patients with no difference between the two treatment arms at any time point (Table 2). During this plateau phase parasite densities estimated by uPCR ranged from 100 to 100,000/mL with median values between 1000 and 10,000/mL (Fig. 1). No significant differences between patients who received additional fish oil and those who did not were seen (see Additional file 1).

**Table 2:** Residual parasite densities (estimated by ultrasensitive PCR quantitation) by treatment arm presented as a percentage relative to baseline density

Time point	AL3		AL5		*p* value^#^
	n/N	(Median; min–max)	n/N	(Median, min–max)	
At 3 days	61/75	0.39 (0–2.72.17)	60/72	0.72 (0–133.48)	0.42
At 5 days	10/20	0.01 (0–82.13)	8/18	0 (0–0.40)	0.50
At 7 days	41/74	0.003 (0–7.00)	40/71	0.003 (0–6.72)	0.72
At 14 days	15/54	0 (0–0.04)	16/55	0 (0–0.44)	0.58
At 21 days	5/52	0 (0–0.01)	8/53	0 (0–0.48)	0.33

*N* number of patients tested, *n* number positive

Residual parasite density % calculation = 100 − [{(Parasite density day 0 − Parasite density day x)/Parasite density day 0} × 100]

^#^Wilcoxon rank-sum (Mann–Whitney) test

#### Prevalence of *Plasmodium falciparum* kelch13 mutations

Of the 154 patients’ samples taken at enrollment, the *k13* gene was sequenced successfully in 152 (98.7%) of which 73 (48%) had a *k13* mutation. There was no difference in *k13* mutation prevalences between the 3 and 5 days treatment groups (48.7% (37/76) vs 47.4% (36/76), p = 0.87). Seventy (96%) of the mutations were concentrated within propeller blades 1–4 in the *k13* gene. Fourteen patients had parasites with a ‘confirmed’ artemisinin resistance mutation according to the WHO classification: C580Y (8), R561H (6), while 38 patients carried parasites with so-called ‘associated’ artemisinin resistance mutations F446I (20), G449A (9), P553L (5) P441L (3), and P574L (1).

#### Determinants of parasite clearance

**Fig. 2:**
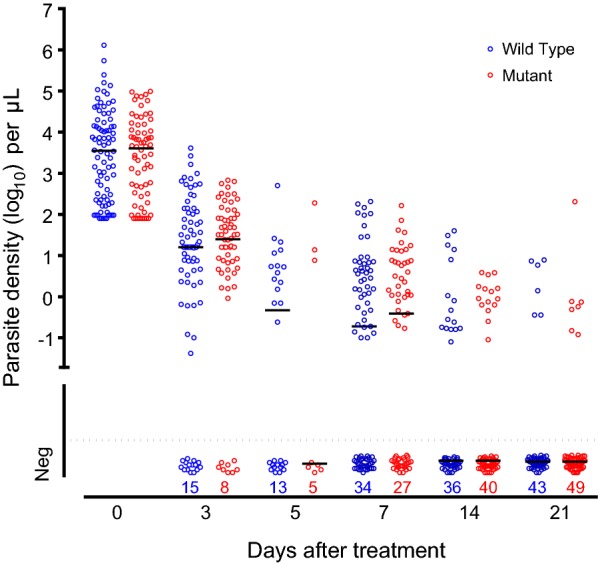
Clearance of *P. falciparum* parasitaemia assessed by quantitative uPCR. One circle represents one patient, red circles are infections with kelch13 mutations after position 440, blue circles represent infections with kelch wild types alleles

Of the 31 patients who were slide positive on Day 3, 52% (16/31) had *k13* mutations (p = 0.49). On multivariable logistic regression > 1% parasitaemia (23/154) on admission was significantly associated with day 3 blood smear positivity (OR: 6.9 [95% CI 2.6–18.4], p < 0.001), whereas infection with a *k13* mutation was not (p = 0.25). *k13* mutations did not affect the slow sub-microscopic phase of parasite clearance; at 21 days after treatment, 12.1% (7/58) patients with the *kelch13* propeller mutation had detectable parasitaemia by uPCR compared to 12.8% (6/47) of those with wild type (p = 0.91) (Fig. 2). The proportions of patients with uPCR detectable parasitaemia at day 21 were similar in the two treatment groups; 10% (5/52) for AL3 and 15% (8/53) for AL5. Thus there were no significant effects of either *k13* mutations or duration of treatment on uPCR positivity at 21 days after treatment (p > 0.5) (see Additional file 1).

#### Gametocytaemia

Gametocytaemia was detected by microscopy on admission in 17 of 78 (22%) of the AL3 arm and 16 of 76 (21%) of the AL5 arm (Table 1). On day 3 these proportions were 8% (6/75) and 13% (9/72), respectively. By day 7 only 1 patient (AL5 group) had gametocytaemia (1.4% [1/71]). In two patients gametocytaemia was detected on day 3 but not on admission, one after AL3 and one after AL5. In both cases gametocytaemia had cleared by day 5.

#### Haematological changes and adverse events

The mean (SD) haemoglobin concentrations on admission in the AL3 and AL5 treatment arms were 12.2 (1.9) g/dL and 12.0 (2.2) g/dL respectively (p = 0.656). After 28 days of treatment, the mean (SD) haemoglobin values were 12.4 (1.7) g/dL for the 3-day and 12.5 (1.6) g/dL for the 5-day treatment arms.

A total of 35 adverse events were reported, 18% (14/78) for AL3 and 13% (10/76) for AL5. There was no significant difference between the two treatment regimens (p = 0.39). Dizziness was the most commonly reported adverse event (n = 9; 4 for AL3 and 5 for AL5), followed by headache (n = 8; 4 for AL3 and 4 for AL5). All adverse events were only of mild or moderate severity and all patients recovered fully. No serious adverse events were reported.

## Discussion

Artemisinin resistance in *P. falciparum* has spread across mainland South-East Asia and led to the failure of ACT in the eastern Greater Mekong subregion and along the Thailand-Myanmar border. There is natural concern over the continued efficacy of ACT in Myanmar, which bears the greatest burden of malaria in the region. However, in this study conducted in Eastern Myanmar the effectiveness, and thus the efficacy of the current first-line treatment artemether–lumefantrine was excellent, despite a high prevalence of the *k13* mutations which are associated with artemisinin resistance. These results are similar to those of a previous study in Myanmar conducted in 2009 (AL3; 28 day 96.8% and 63-day 90.3%) [27]. Artemether–lumefantrine has been the national protocol for falciparum malaria in Myanmar since 2002 and has been used extensively, particularly in the last few years. Although these results are reassuring for the present they should not give rise to complacency. Background immunity may have contributed to drug efficacy, and the predominant mutation (F446I) in the study area appears to be associated with slightly faster parasite clearance than other common propeller mutations [26]. Further east parasites bearing the C580Y mutation have predominated, and these have been associated with ACT failure [1, 2, 4, 9].

Although lumefantrine efficacy was still preserved in this location at the time of the study, it is likely that resistance will worsen eventually. The recent reduction in malaria transmission in Eastern Myanmar as a result of active intervention programmes and substantially improved ACT availability will reduce population immunity and this could compromise therapeutic responses. The longer course and thus higher dose of artemether–lumefantrine evaluated here was well tolerated and, if deployed, would be expected to provide greater efficacy if resistance does worsen, and thus prolong the useful therapeutic life of this important anti-malarial drug. In general anti-malarial drug policy changes occur late in the course of resistance emergence, and the delays result in increased transmission, increased morbidity, and compromise of the opportunity to modify the dose regimen to maintain efficacy. In Thailand in the early 1990s mefloquine resistance was already far advanced before the dose was increased from the original 15 mg/kg to the now generally accepted 25 mg/kg dose [28–30]. The increased efficacy lasted only a few years. If the higher dose had been introduced earlier, the useful therapeutic life might have been prolonged. Similar considerations may apply to lumefantrine in Myanmar [28].

Highly sensitive PCR methods (uPCR) allow quantitation of parasitaemia at densities one thousand times lower than microscopy [24, 31]. This new approach to assessing the therapeutic response was implemented in this effectiveness study in order to characterize the sub-microscopic parasite clearance dynamics associated with artemisinin resistance. It revealed a biphasic pattern of parasitaemia decline with a slower second phase at parasite densities close to the limit of microscopy detection. The initial phase represents the parasite counts usually measured in treatment studies from which parasite clearance kinetics are conventionally derived. This phase was not characterized in this study, but large studies elsewhere show clearly that this initial phase is significantly slowed in the presence of k13 mutations, reflecting the in vivo phenotype of artemisinin resistance [1]. In vitro this reflects reduced ring stage susceptibility [32, 33]. However in contrast the second slower phase captured in this study was similar in character and magnitude in wild type *k13* parasites (artemisinin sensitive) and *k13* mutant parasites (artemisinin resistant), and it was not affected by length of treatment. Persistent low density gametocytaemia is one possible explanation, but a single gametocytocidal dose of primaquine was given to all patients at the start of the treatment. Primaquine is rapidly gametocytocidal and this results in rapid clearance of gametocyte mRNA [34]. It therefore seems unlikely the continued DNA signal in this study results from slow clearance of the sexual stages. The most likely explanation is that this represents the sub-population of dormant parasites. Dormancy remains a poorly understood yet well-recognized phenomenon which is thought to account for recrudescence following 7 day courses of artemisinin derivatives [35, 36]. It has also been suggested that artemisinin resistance may be associated with dormancy [35] although in induced resistance to the semisynthetic artemisinin derivative artelinic acid, resistance was associated with a reduced propensity to dormancy [36]. However in this study the dormant fraction, which was substantially less than 0.1% of the pretreatment parasites appeared to be independent of artemisinin resistance. Importantly despite persistence for up to 3 weeks these parasites did not cause recrudescence. Presumably residual lumefantrine levels were sufficient to kill them if and when they “awoke”. Further parasitological research and modelling is underway to try and determine the origin, biological state and kinetics of this slow clearing parasite nucleic acid.

Although almost half the falciparum malaria infections in this region of Myanmar bore *k13* mutations in the south-eastern part of Myanmar, the effectiveness of the current first-line treatment of 3 days artemether–lumefantrine remained high. This may be explained, at least in part, by the predominance of the F446I *k13* mutation causing an intermediate resistance phenotype [26]. How long this will last is uncertain.

## Conclusion

If more resistant *k13* mutations, such as the C580Y mutation, in a “fitter” genetic background take over, as they have further east [5, 6], then partner drug resistance and reduced efficacy may follow as has occurred in Cambodia and neighbouring countries. The 5 day artemether–lumefantrine treatment course was well-tolerated and could be considered as a strategy to pre-empt development of resistance to lumefantrine in East Asia.

## Additional file

**Additional file 1.** Contains trial profile, Additional figures, Additional results tables, and a summary of ultrasensitive PCR quality control procedures.
