# Supplementary material for: Effectiveness and safety of 3 and 5 day courses of artemether–lumefantrine for the treatment of uncomplicated falciparum malaria in an area of emerging artemisinin resistance in Myanmar
Source: Malar J. 2018 Jul 11;17:258. doi: 10.1186/s12936-018-2404-4 (PMC6042398; doi:10.1186/s12936-018-2404-4)
Supplement: Supplementary file 1 — Additional file 1. Contains trial profile, Additional figures, Additional results tables, and a summary of ultrasensitive PCR quality control procedures. [file 12936_2018_2404_MOESM1_ESM.docx]

Additional File 1 (Tun *et al*)

Discontinued (n=5)

- Withdrew consent (n=2)
- Lost to follow up(n=3)

Discontinued (n=8)

- Withdrew consent (n=4)
- Lost to follow up (n=4)
- uPCR follow up on Day 3, 5, 7, 14 and 21
- uPCR follow up on Day 3, 5, 7, 14 and 21

Assessed for eligibility (n=1311)

Excluded (n=1157)

♦ Not malaria (n=1054)

♦ Declined to participate (n=3)

♦ Other malaria (*Pv, Pm*) (n=67)

♦ Signs of severe malaria (n=2)

♦ *Pf* parasite counts too high or too low (n=16)

♦ Other reasons (n=15)

3 Day AL (n=78)

5 Day AL (n=76)

- Completed at day 42 without recurrence (n=70)
- Failure on or before day 42 (n=0)
- Completed at day 42 without recurrence (n=69)
- Failure on or before day 42 (n=2)

Additional Figure S1. Trial profile

Additional Figure S2. uPCR positivity for both treatment arms from Day 0 to Day 21.

Additional Figure S3. Effect of taking fish oil together with the anti-malaria treatment on *Plasmodium falciparum* positivity

Additional Figure S4. Primary amino acid positions of *P. falciparum* kelch13 mutations in Kyainseikgyi Township, Kayin state, Myanmar

Additional Figure S5. Kaplan-Meier survival curves of kelch13 propeller mutations versus wild type

Additional Table S1. Relationship between blood smear day 3 positivity and uPCR positivity during follow up

| Time point | Patients microscopy Positive on day 3 | Patients microscopy Negative on day 3 |
| --- | --- | --- |
|  | uPCR positive (%) | uPCR positive (%) |
| Day 3 | 30/31 (97.0) | 91/116 (78.0) |
| Day 5 | 8/9 (91.0) | 10/29 (34.0) |
| Day 7 | 26/30 (87.0) | 55/114 (48.0) |
| Day 14 | 9/21 (43.0) | 22/86 (26.0) |
| Day 21 | 4/20 (20.0) | 9/85 (11.0) |

Additional Table S2. Effect of taking fish oil together with the anti-malaria treatment on *Plasmodium falciparum* positivity

|  | uPCR positivity after AL treatment without fish oil  positives/total (%) | uPCR positivity after AL treatment with fish oil  positives/total (%) |
| --- | --- | --- |
| On admission | 77/77 (100%) | 77/77 (100%) |
| 3 day after treatment | 61/74 (82.4%) | 60/73 (82.2%) |
| 7 day after treatment | 39/73 (53.4%) | 42/72 (58.3%) |
| 14 day after treatment | 13/48 (27.8%) | 18/61 (29.5%) |
| 21 day after treatment | 9/46 (19.6%) | 4/59 (6.8%) |

Additional Table S3. Artemether-lumefantrine dosing table

| Body weight in kg | No. of tablets recommended at approximate timing of dosing | | | | | |
| --- | --- | --- | --- | --- | --- | --- |
|  | 0 h | 8 h | 24 h | 36 h | 48 h | 60 h |
| 5–14 | 1 | 1 | 1 | 1 | 1 | 1 |
| 15–24 | 2 | 2 | 2 | 2 | 2 | 2 |
| 25–34 | 3 | 3 | 3 | 3 | 3 | 3 |
| >34 | 4 | 4 | 4 | 4 | 4 | 4 |

One tablet of AL contains 20mg artemether and 120mg lumefantrine. Patients receiving the 5 day course continued the same daily dose for 2 additional days.

Additional Table S4. Positivity rates and hazard ratios for risk factors of uPCR positivity at 21 days after treatment

| Risk factor |  | Percentage uPCR positive | Hazard Ratio (95%CI) |
| --- | --- | --- | --- |
| Treatment arm | AL3 | 10 (3 to 21) | 1.1 (0.74 to 1.51) |
|  | AL5 | 15 (7 to 28) |  |
| Kelch13 | WT or <440 | 13 (5 to 26) | 0.89 (0.62 to 1.28) |
|  | Mutation >440* | 12(5 to 23) |  |

*Artemisinin resistance was defined by a parasite clearance half-life longer than 5 hours, with some Pfkelch13 polymorphisms (beyond amino acid position 440).

Additional Table S5. Gametocyte carriage among patients with gametocytemia on admission

| Day | AL3 | AL5 |
| --- | --- | --- |
| 0 | 17/78 (21.8%) | 16/76 (21.1%) |
| 3 | 6/75 (8.0%) | 9/72 (12.5%) |
| 5 | 0/75 (0%) | 2/73 (2.7%) |
| 6 | 0/75 (0%) | 1/73 (1.4%) |
| 7 | 0/74 (0%) | 1/71 (1.4%) |
| 14 | 0/74 (0%) | 0/72 (0%) |

Summary of quality control procedures for qPCR assay:

1. Contamination precautions were rigorously applied in the laboratory. Checking for contamination was performed by routine random insertion of known negative samples (15% of total) in each run. If a negative control yielded a positive result in routine testing, the whole run was repeated and retested until all negative control gave a negative reading (invariably in the next run). Root cause analysis usually identified the concentrated positive control as the source of contamination. Investigation of potential contamination involved genotyping based on the *msp1*, *msp2*, and *glurp* genes for *P. falciparum* and on the microsatellites markers pv3.27, pv3.502, pv ms5 for *P. vivax*, following previously published protocols for *P. falciparum* (1) and *P. vivax* (2).
2. The primers and PCR protocol were optimized to proper conditions, no non-specific band/ primer-dimer was found.
3. The possibility that cell free malaria DNA could cause false positives was considered. Malaria DNA in merozoites is released from parasitized red cells at the moment of schizont rupture, and free merozoite DNA persists in the circulation. Data from clinical studies indicated that free DNA would only be a significant confounder if recent parasitaemias were in the range detectable by microscopy, and plasma comprised > 10% of the sample. In this study the plasma of each sample was carefully removed, and only packed red cells were used during DNA extraction. For this reason it is very unlikely that circulating free DNA in plasma is a source of false positive results.

To check for false positive results, we tested

1. 100% of positive uPCR were then tested for speciation using species-specific qpcr. The results confirmed in the positive samples.
2. 20% of positive PCR product were confirmed in agarose gel electrophoresis, they shown a single band with the correct-sized PCR products. Moreover, 10% of PCR product was then performed direct sequencing and confirmed 18sRNA gene amplification. All confirmed.
3. In order to rule out cross-contamination of samples during DNA extraction or PCR processing, negative controls consisting of only water were added in a proportion of 8 for 48 samples. No evidence of cross-contamination of samples in the negative controls of these experiments.

References

1.Anderson TJ, Su XZ, Bockarie M, Lagog M, Day KP. 1999. Twelve microsatellite markers for characterization of Plasmodium falciparum from finger-prick blood samples. Parasitology 119 ( Pt 2):113-125.

2.Imwong M, Nair S, Pukrittayakamee S, Sudimack D, Williams JT, Mayxay M, Newton PN, Kim JR, Nandy A, Osorio L, Carlton JM, White NJ, Day NP, Anderson TJ. 2007. Contrasting genetic structure in Plasmodium vivax populations from Asia and South America. International journal for parasitology 37:1013-1022.
